# Eight-Year Study of *Haemogregarina stepanowi* Infection in Poached European Pond Turtles (*Emys orbicularis*) Held in Belgrade Zoo Quarantine

**DOI:** 10.3390/ani13152429

**Published:** 2023-07-27

**Authors:** Sanja Aleksić-Kovačević, Miloš Vučićević, József Özvegy, Stefan Jelisić, Biljana Djurdjević, Jasna Prodanov-Radulović, Milan Došenović, Darko Marinković

**Affiliations:** 1 Faculty of Veterinary Medicine, University of Belgrade, 11000 Belgrade, Serbia; skovacevic@vet.bg.ac.rs (S.A.-K.);; 2 Belgrade Zoo, 11000 Belgrade, Serbia; 3 Scientific Veterinary Institute “Novi Sad”, Department of Epizootiology, Clinical Diagnostics and DDD, 21000 Novi Sad, Serbia; biljana@niv.ns.ac.rs (B.D.); jasna@niv.ns.ac.rs (J.P.-R.)

**Keywords:** European pond turtle, *Haemogregarina stepanowi*, Serbia, zoo quarantine

## Abstract

**Simple Summary:**

The concept of this investigation emerged when a rapidly increased number of poached European pond turtles in poor health condition, with shell necrosis and massive skin hemorrhages, were temporarily put into a pond that belongs to a quarantine section at Belgrade Zoo. Using cytology as an initial tool, different parasitic stages of hemogregarines have been noticed in the blood smears of the examined animals. Cytological and microscopic examination of the samples proved to be sufficient for establishing the infection, but molecular analyses were used in examining the phylogeny of the blood parasites. Massive hemorrhages in the skin, as well as shell necrosis, were the most prominent findings observed in the turtles. In addition, the lungs, liver, kidneys, and spleen revealed hyperemia and massive hemorrhages, along with the presence of parasitic stages of *Haemogregarina stepanowi* in tissue samples. Furthermore, the reduced hematocrit value found in the examined population of infected turtles indicated anemia. Over the eight years (2015–2023) of monitoring, the number of diseased and dead turtles has decreased, which could be hypothetically attributed to the elimination of leeches as the definitive host.

**Abstract:**

The eight-year study (2015–2023) was performed on a large sample of poached European pond turtles infected with *Haemogregarina stepanowi* and held in a pond that belongs to a quarantine section of Belgrade Zoo. The protected species of European pond turtles have been found in poor health, with general weakness, anorexia, and low motility. Comprehensive cytological, hematological, molecular, and postmortem evaluations have been performed. Initially, Diff Quick staining of the blood smears revealed rounded or elongated erythrocytes, often bearing premeront or U-shaped gamont of the hemogregarines inside. The reduced erythrocyte numbers, hemoglobin, and hematocrit values found in the examined population of infected turtles indicated anemia. Macroscopically, shell necrosis and massive skin hemorrhages were the most prominent findings observed in diseased turtles. Microscopically, the lungs, liver, kidneys, and spleen revealed hyperemia, hemorrhages, and the presence of parasitic stages in tissue samples in 31 of 40 necropsied turtles. Cytological and microscopic examination of the samples proved to be sufficient for establishing the infection, but molecular analyses of the 18S sequence were used for phylogenetic studies. Over the years, the number of diseased and dead turtles has decreased, which could be hypothetically attributed to the elimination of leeches as the definitive host.

## 1. Introduction

The European pond turtle (*Emys orbicularis*) is a protected species of water turtle widespread in natural habitats in Serbia. Diagnosing and identifying the causative agent in diseased turtles is related to their specific body structure and hidden way of life in nature [1]. On the other hand, captive turtles could be exposed to feeding disorders, overcrowding, and insufficient vector control, which affects the health condition often with non-specific clinical symptoms. Therefore, it is necessary to apply a protocol that includes different diagnostic methods [2,3].

The blood parasites of pond turtles are adapted to variations in reproductive and migratory habits, and infected turtles usually show poor health conditions and low motility [4]. Despite the initial importance of cytological diagnosis of hemogregarines, molecular methods enable the detection and identification of parasites in the sample and also an investigation of evolutionary relationships of parasites within the Apicomplexa [5,6,7]. Genus *Haemogregarina* Danilewsky, 1885 (Phylum Apicomplexa, Class Sporozoasida, Order Adeleina, Suborder Adeleorina, Family *Haemogregarinidae*), infects lower vertebrates as intermediate hosts and the leeches as definitive hosts. An infected leech could inject merozoites into the bloodstream of turtles [8], lizards [9,10], and crocodiles [11], initiating the merogony in the host’s lungs, liver, and spleen, followed by secondary merogony, which takes place in the erythrocytes [8,12,13]. Different parasitic stages of hemogregarines are usually identified during the examination of the blood smear within the erythrocytes [14].

The concept of this eight-year-long research was originally started in 2015 when a rapidly increased number of diseased poached European pond turtles (*Emys orbicularis*) were temporarily kept in a pond that belongs to the quarantine section of the Belgrade Zoo. The majority of animals have been found in poor health, with shell necrosis as well as massive skin hemorrhages. After the primary results of cytological and hematological analyses showed the presence of blood parasites and anemia, a clinical examination of all turtles with general weakness was undertaken. We faced the opportunity for valuable cytological, hematological, molecular, and pathological investigations on a large number of diseased animals. This comprehensive study, including monitoring of infection dynamics during the eight-year period (2015–2023) in captive turtles, has not been carried out so far. Due to the scarce morphological descriptions in the literature related to hemogregarines in turtles, our study highlights pathological findings in different organs in a large sample of European pond turtles infected with *Haemogregarina stepanowi*. Bearing in mind that identifying the causative agent in diseased turtles in natural habitats is not easy due to their hidden way of life, our results of postmortem investigation could be helpful in the future for obtaining a preliminary diagnosis. Also, the results of the morphological evaluations have not been previously published, which brings considerable value and novelty to this work. In addition, regarding infection dynamics, we have monitored the presence of leeches as vectors for hemogregarines, taking into account the factors that led to the almost complete disappearance of the infection over time.

## 2. Materials and Methods

### 2.1. Animals

All European pond turtles (*Emys orbicularis*) as protected animal species were examined in compliance with the national law on care, and all ethical requirements were considered.

European pond turtles investigated in the current study were poached, seized in transit in 2015, and temporarily placed in a pond with a natural inflow of water in the quarantine section of the Belgrade Zoo. At the same time, no other diseased turtles were present in the same pond of the quarantine section. Approximately 100 turtles have been found with general weakness, poor mobility, and anorexia. The first group of thirty (15 male and 15 female) turtles selected for investigation in 2015/2016 were marked with unique identification numbers, and the blood samples were taken for cytological, hematological as well as molecular analyses. The necropsy was performed on all 30 turtles that died over time within this group. Detailed macroscopic and microscopic postmortem findings were recorded, and the images were saved.

Considering that comprehensive targeted trials were prolonged continuously in 2018/2019, the second group of 9 (6 male and 3 female) European pond turtles was selected based on evidence of low motility, and they were clinically evaluated. The blood samples were taken for cytological and hematological analyses as well as the molecular identification of parasites whose presence was determined in the previously examined group of turtles. Also, in the same temporal group, 6 dead turtles were necropsied (4 male and 2 female) and subjected to postmortem evaluation.

Continuing monitoring in 2022/2023, the third group of 16 turtles (10 male and 6 female) was selected for the blood sampling procedure, although without evidence of weakness, anorexia, or other clinical signs. Cytological, hematological, and molecular analyses were done for each of the 16 turtles. The necropsy and postmortem evaluation were performed on four animals (2 male and 2 female) which were found dead after hibernation. Additionally, the blood samples of six clinically healthy turtles (two in each of the three mentioned phases of investigation) from the other pond were sampled for the same panel of analysis for control purposes. Briefly, the other pond serves as a regular habitat for healthy turtles, which are open daily to zoo visitors.

### 2.2. Clinical Examination

The turtles selected for the study were marked for identification and measurements of the length, width of the carapace and plastron, as well as height and body mass. Clinical examination included inspection, and evaluation of the turtle’s habitus and mobility, followed by detailed observation of the skin (head, limbs, and tail) as well as examination of the plastron and carapace. In particular, hemorrhagic spots were observed in the axillary and inguinal regions. In addition, the presence of leeches on the turtle’s body, anorexia, dehydration as well as assessment of the general condition of the animals was recorded.

### 2.3. Blood Sampling, Hematological, and Cytological Analyses

Blood samples (2–3 mL) from all European pond turtles (*E. orbicularis*) were collected from the subcarapacial venous sinus using 23 G × 1 Luer needles. Air-dried (18–20 °C for 1 h) blood smears were fixed and stained with Diff Quick (Hemacolor^®^ Merck, Rahway, NJ, USA). Stained slides were examined with the immersion objective on an Olympus BX51^®^ microscope (Olympus, Tokyo, Japan), and images were recorded using an Olympus Color View III^®^ digital camera (Olympus, Japan). Morphological identification of forms of the blood parasites was performed based on Telford’s classification [15]. Due to the morphology of erythrocytes in turtles, manual laboratory methods (modified Neubauer chambers for cell counting) were used. Hematocrits were measured using microhematocrit tubes after centrifugation for 5 min at 7000 RPM.

### 2.4. Molecular Investigation (PCR)

The molecular study was carried out in the Department of Biology, Faculty of Veterinary Medicine, University of Belgrade.

For DNA extraction, 10 μL of whole blood was collected from each turtle. The “KAPA Express Extract Kit” (Cat. No. KK7152, Kapa Biosystems, Cape Town, South Africa) was used for extraction according to the manufacturer’s instructions.

PCR amplification was performed with specific primers (EF: 5′-GAAACTGCGAATGGCTCATT-3′ and ER: 5′-CTTGCGCCTACTAGGCATTC-3′) described by Kvičerová et al. (2008) [16]. These primers targeted fragments up to 1500 bp in length of the 18S rRNA gene sequence of hemogregarins. The PCR reaction mix was prepared using the KAPA2G Robust HotStart ReadyMix (Cat. No. KK7152, Kapa Biosystems, Cape Town, South Africa) according to the manufacturer’s instructions. Amplifications were performed using the MultiGene Gradient PCR thermal cycler (Labnet International Inc., Edison, NJ, USA). The thermal protocol consisted of an initial denaturation step at 95 °C for 4 min, followed by 35 cycles of denaturation (95 °C, 30 s), annealing (58 °C, 30 s), and DNA extension (72 °C, 90 s). The final extension step was performed at 72 °C for 10 min. A DNA isolate of *Haemogregarina stepanowi* from the authors’ collection served as a positive control for PCR, while DNA/RNA-free water was used as a negative control.

The PCR products obtained were directly sequenced in both directions using the BigDye^®^ Terminator method on an ABI 3730XL DNA sequencing machine (Macrogen Europe, Amsterdam, The Netherlands). Sequence similarity analysis was performed using BioEdit version 7.2.5 [17] and the software Clustal W.

### 2.5. Pathomorphological Examination

Pathomorphological examination was conducted at the Department of Pathology, Faculty of Veterinary Medicine, University of Belgrade. Necropsy was performed on a total of 40 pond turtles from monitored groups: thirty turtles (2015/2016), six turtles (2018/2019), and four turtles (2022/2023). After macroscopic examination, the selected tissue samples (skin, lungs, spleen, liver, kidney) were fixed for 48 h in 10% neutral buffered formalin. They were embedded in paraffin blocks after standard processing in an automatic tissue processor. The paraffin sections (3–5 µm) were stained with hematoxylin-eosin (HE). Stained slides were examined on an Olympus BX51^®^ microscope (Olympus, Japan), and images were recorded using an Olympus Color View III^®^ digital camera (Olympus, Japan).

## 3. Results

### 3.1. Clinical Examination

Hemogregarines were present in blood smears of all 30 European pond turtles examined from the first group in 2015/2016. The all-infected turtles were found in poor condition, with massive skin hemorrhages mostly in the inguinal region and, in some cases, with severe shell necrosis on the plastron.

Clinical investigation of nine European pond turtles from the second group evaluated in 2018/2019 revealed small focal shell necrosis in two male turtles as well as low motility and anorexia in two females. However, hemogregarines were not present in the blood of any turtle from this group, nor in six dead animals.

The 16 European pond turtles from the recently evaluated group in 2022/2023 did not exhibit any clinical signs, except for six turtles with low motility and pale appearance of the oral cavity mucosa. However, only one of them showed the presence of hemogregarines in blood smears, but all four necropsied turtles from this temporal group were negative for hemogregarines.

During the monitoring of the pond, leeches were frequently found in 2015/2016 but not in 2018/2019 and 2022/2023. Additionally, they were never detected on the turtle’s body of any monitored group.

### 3.2. Blood Smears Cytology

**Figure 1:**
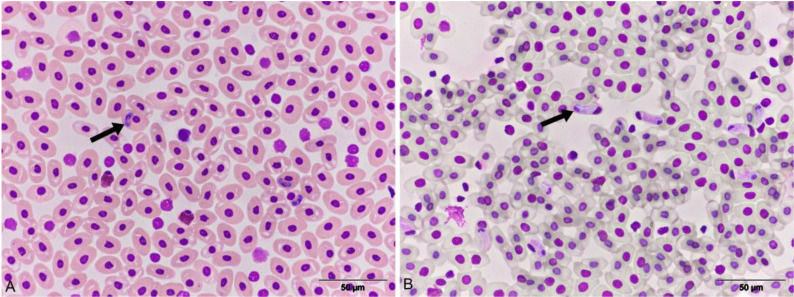
European pond turtle, blood smear, (**A**) premeront of the *Haemogregarina* sp. in erythrocytes, (arrow) DQ × 600; (**B**) Gamont of the *Haemogregarina* sp. in erythrocytes, (arrow) DQ × 600.

In the blood smears collected from a total of 30 animals during the years 2015 and 2016, as well as from a single turtle in 2022 and 2023, notable alterations in the shape and size of infected erythrocytes were observed. These infected cells often exhibited a rounded or elongated appearance, and some displayed a curved or pear-shaped morphology with nuclei that appeared eccentric and atrophic. Within the blood smears, the presence of both premeront (Figure 1A) and U-shaped gamont (Figure 1B) of the *Haemogregarina* sp. were identified within the erythrocytes, as per the classification method established by Telford [15]. Conversely, the blood samples collected from six turtles in the control group, which were in clinically healthy condition, did not reveal any infected erythrocytes.

### 3.3. Hematological Analysis

Hematological analysis of infected pond turtles showed decreased hematocrits and numbers of red blood cells as well as low hemoglobin levels. The parameters and values originating from six control clinically healthy turtles were within the reference range (Table S1).

### 3.4. Molecular Investigation (PCR)

Amplification of the 18S rRNA sequence was observed in all 30 turtles of the first group (2015/2016). However, none of the nine turtles in the second group (2018/2019) showed amplification, and only one of the 16 turtles in the third group (2022/2023) showed amplification. All PCR products were the same size as the amplicon of *H. stepanowi*, which served as a positive control. In addition, the 18S rRNA sequences from our study were identical in all samples [GenBank Accession Number KT749877; http://www.ncbi.nlm.nih.gov, accessed on 15 April 2023] and had 100% nucleotide similarity to the 18S rRNA gene sequence of *H. stepanowi* [GenBank Acc. No KF257927; http://www.ncbi.nlm.nih.gov, accessed on 15 April 2023]. No amplification was observed in the control group, which consisted of six clinically healthy turtles.

### 3.5. Pathomorphological Findings

#### 3.5.1. External Postmortem Evaluation

**Figure 2:**
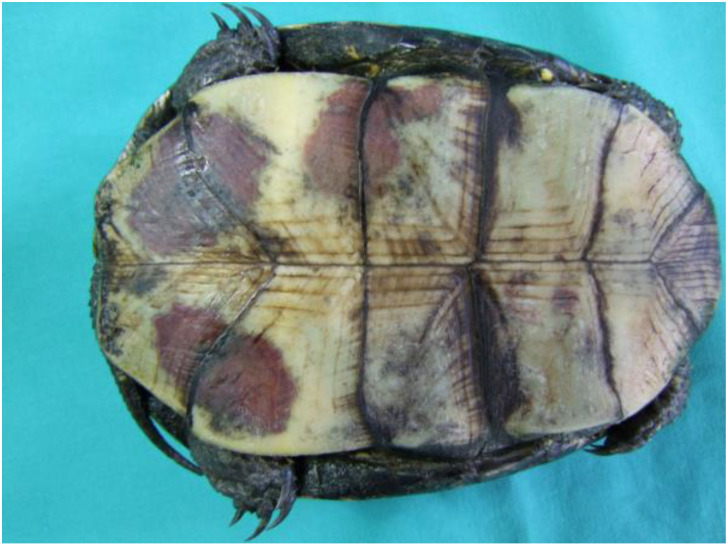
European pond turtle. Diffuse massive hemorrhages on the plastron are most prominent in the abdominal, femoral, and anal scutes [18] (according to Hernandez-Divers et al., 2009).

Macroscopic changes on the shell were observed in 73.3% of infected turtles but more frequently on the plastron than on the carapace. The majority of shell lesions were in the form of erosions or up to 2 mm deep necrosis. Deformation and lack of bony plates on the carapace as well as on the plastron were found in two turtles. Diffuse, severe, dark-red, well-bounded hemorrhages on the plastron were the most prominent at the abdominal, femoral, and anal scutes, as well as the connections between the carapace and the plastron. These lesions were observed in 23.3% of turtles (Figure 2).

**Figure 3:**
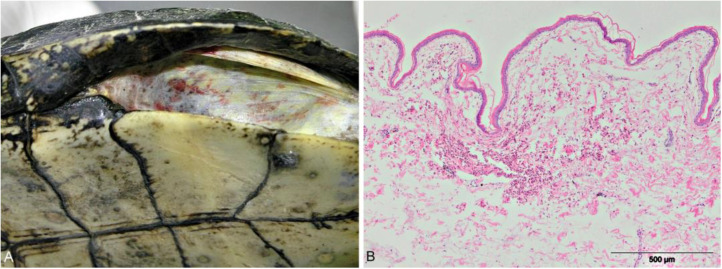
(**A**) European pond turtle. Diffuse massive hemorrhages on the skin; (**B**) Diffuse hemorrhages in the epidermis, HE × 100.

Macroscopic examination showed massive skin hemorrhages in 76.6% of pond turtles, mainly in the inguinal and axillary regions (Figure 3A). Microscopically, diffuse hemorrhages were observed in the epidermis (Figure 3B). The oral cavity mucosa was often pale and revealed anemia in turtles positive for *Haemogregarina stepanowi*.

#### 3.5.2. Internal Postmortem Evaluation

Lungs: After removing the carapace in 93.3% of the examined turtles, a clear yellowish liquid (3–5 mL) was present, which corresponds to hydrocoelom. Microscopic analysis of lungs revealed hyperemia, hemorrhage, and hyperplasia of the bronchiolar goblet cells, followed in some cases by mucinous bronchiolitis as well as the presence of *Haemogregarina* sp. in two turtles.

Kidneys: Enlarged kidneys with multifocal hemorrhages were observed in 26.6% of diseased turtles. Microscopically, interstitial nephritis was present in the kidneys of 46.6% of turtles as well as moderate subcapsular hemorrhages with hemosiderosis and the presence of *Haemogregarina* sp. in erythrocytes in five cases.

Spleen: In 53.3% of turtles, the trabecular sinuses of the spleen were often enlarged, hyperemic, and filled with numerous melanomacrophages. The blood parasite *Haemogregarina* sp. was observed in the spleen of seven turtles.

**Figure 4:**
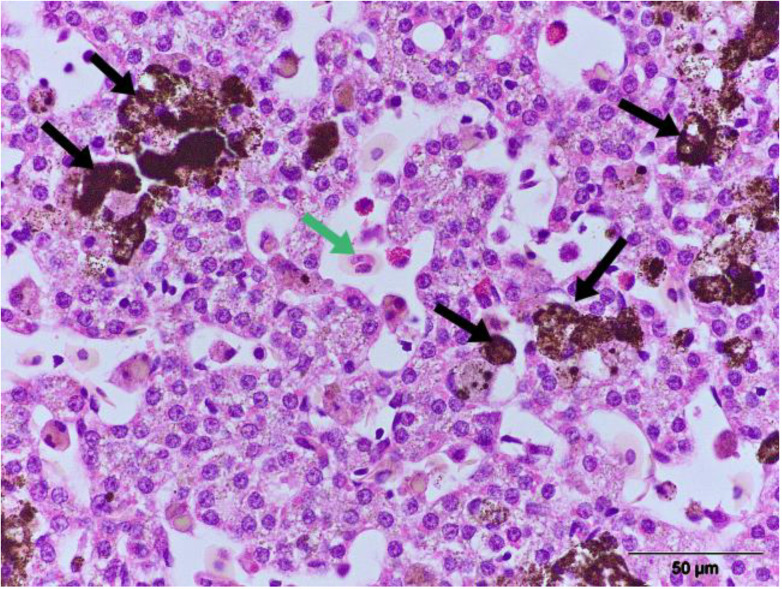
European pond turtle. Liver. The presence of numerous melanomacrophages (black arrow) and *Haemogregarina* sp. (green arrow) (HE × 600).

Liver: The fibrous liver capsule of infected pond turtles was generally smooth and shiny. However, one-third of turtles exhibit multifocal (1–2 mm) lesions on the surface of the liver, microscopically composed of lymphocytes as well as granulocytic inflammatory infiltrates. In five cases, numerous melanomacrophages and the presence of *Haemogregarina* sp. were observed (Figure 4).

## 4. Discussion

Captive reptiles are often exposed to inappropriate temperatures, nutritive deficiencies, and insufficient vector control, which play an important role in blood parasite transmission. Reptiles of natural habitats could also host hemoparasites, including hemogregarines, hemococcidians, and hemosporidians [15,19].

The establishment of molecular techniques for the detection of blood parasites [20] increased the knowledge of apicomplexan parasite diversity in reptiles worldwide [7,21,22]. Molecular investigation in pond turtles from the Belgrade Zoo quarantine has confirmed the presence of *Haemogregarina stepanowi* (a member of the Family *Haemogregarinidae*, Phylum Apicomplexa). Apicomplexan cells have been recently described as cells with elongated mitochondria and unique apicoplast organelles. Molecular phylogenies have been based on nuclear, mitochondrial, and apicoplast DNA sequences [23]. The most recently published data indicate the presence of different lineages of *Haemogregarinae* in *E. orbicularis* obtained from seven different localities in Serbia and North Macedonia [24]. However, all animals monitored in our investigation were derived from the same natural habitat, and they were kept in the same pond in the quarantine section of Belgrade Zoo, which could explain the detection of the amplifications of 18S rRNA sequence in all 31 positive animals. Further phylogenetic analyses were not included in this work.

*Haemogregarina stepanowi* has a wide range of distribution in Europe, Turkey, and the Middle East. However, no previous work has been conducted on hemogregarines in captive European pond turtles (*Emys orbicularis*) in Serbia. The comprehensive eight-year study (2015–2023) included European pond turtles, which were found in poor health conditions, with anemia, general weakness, and anorexia. Using cytology as an initial tool, *Haemogregarina* sp. has been noticed in the blood smears of 31/55 evaluated animals.

*Haemogregarina stepanowi* occurs in several shapes depending on the phase of the life cycle [15]. In our study, cytological analysis of blood smears in turtles showed the presence of intraerythrocytic premeronts and gamonts of the hemogregarinas. Infected erythrocytes were rounded, elongated, curved, and pear-shaped with eccentric and atrophic nuclei. Intraerythrocytic premeronts were oval-shaped and had centrally positioned nuclei, while gamonts were U-shaped and recurved into two limbs with nuclei located near the bend of the midgamont. According to data in the literature, trophozoites, which occupy a polar position in erythrocytes, have little or no influence on the shape of the cell and position of the nucleus, but premeronts and gamonts affect the erythrocyte and disposition the nucleus to a polar or lateral side [15].

However, the molecular investigation confirmed *Haemogregarina stepanowi* in 31 of all investigated European pond turtles, although with unequal temporal distribution: the majority (30 individuals) tested positive in 2015/2016 [23], none of 9 tested turtles were found positive in 2018/2019, and only one of 16 recently checked and mostly asymptomatic turtles were positive for *Haemogregarina stepanowi*. Additionally, PCR proved to be a very accurate method for the detection of *H. stepanowi* from whole blood samples of European pond turtles. In all cytological positive samples, the nucleotide sequence of the parasite was found. Also, nucleotide sequencing showed no difference between the sequence obtained from our samples and the sequence reported by Dvořáková et al. [6].

According to current thought, hemogregarines evolved first in invertebrates but then diverged once they incorporated vertebrates into their life cycle [25]. The genus *Haemogregarina* has a low specificity for the vertebrate host, and the distribution is probably related to the vector and the final invertebrate host—the leech [6]. Considering the importance of invertebrate vectors of apicomplexan parasites, the skin of the head, limbs, and tail of each turtle in this study was examined for leeches. However, they were never detected on the turtle’s body of any monitored group. The leeches were frequently found in the pond in 2015/2016, even though over time, their number decreased rapidly during 2018/2019, and finally 2022/2023, there were no more leeches to be found in the pond. We assume that the most important role in eliminating leeches in that habitat is played by the introduction of routine pond cleaning and mechanical removal of the leeches. However, the potential role of the birds (mostly cormorants and egrets) that land in the surroundings of the pond could not be excluded. Nevertheless, the leeches are known as invertebrate hosts and vectors for parasites of aquatic turtles, and ticks are the most common hosts for parasites of terrestrial reptiles [25,26,27]. The absence of infected turtles in our samples (2018/2019) could be explained by the current absence of appropriate aquatic vectors infecting turtles. Additionally, our results obtained in this group should be taken with caution due to the lower sample sizes collected in that period.

In the conducted study, no significant difference in the prevalence of hemogregarines was detected between male and female turtles. However, in some studies, females were found to be more commonly infected with hemogregarines [28]. Additionally, all of the infected turtles were adults, showing that the longer life and bigger size probably promote them as better targets for leech attachment [29].

The different prevalence of hemogregarines in erythrocytes in the current study is similar to other studies in turtles infected with hemogregarines [25,27,28]. In addition, in our study, *Haemogregarina stepanowi*-infected turtles revealed alterations in hematocrit and hemoglobin concentrations. However, similar parameters and values could be common findings in captive reptiles with malnutrition and other chronic conditions [30,31].

The results of our clinical and postmortem evaluation revealed that the majority of infected turtles had shell necrosis and massive hemorrhages. Nevertheless, these changes could be explained as a result of specific environmental conditions in turtles seized in transit through Serbia but also as the consequence of previous challenges in nature, which was already noticed in other studies [1,4,32]. Although postmortem reports are still very limited in the literature [33], in our study, massive skin hemorrhages in the inguinal and axillary regions were the most prominent findings observed in turtles. In addition to skin hemorrhages, the lungs, liver, kidneys, and spleen revealed hyperemia and massive hemorrhages, which could be attributed to other pathological conditions [34]. Having in mind that hemorrhages are common findings in spirorchiidosis in *E. orbicularis* [35], we have no evidence of intravascular trematodes or intracytoplasmic trematode eggs in a broad range of microscopically examined tissues of *E. orbicularis* in the current study. In some cases, haemorrhages caused by ranaviruses may occur in the gastrointestinal tract of aquatic turtles, but they are often the result of secondary infections, so the ranaviruses were not confirmed in some studies [36]. However, some authors reports lack of ranaviruses or herpesviruses in comprehensive studies [37].

Furthermore, multifocal lesions were observed on the surface of the liver in one-third of infected turtles. However, due to the lack of data on postmortem findings, in one report, granulomatous changes in the liver were described [38]. The microscopical evaluation revealed that the structure of the granulomatous lesion was dominated by granulocytes, lymphocytes, and to a lesser extent, macrophages. Considering the role of innate immunity, T cells would contribute to the activation of granulocytes and monocytes/macrophages that have also been shown to recognize parasite materials. However, more studies with higher sample sizes are needed to understand the host cell response during the hemogregarines infection. The advanced knowledge of this information can be critical to understanding the morphological changes as well as cell and tissue responses to the hemogregarines presence in vulnerable and often endangered host groups such as European pond turtles.

## 5. Conclusions

This comprehensive study expands the knowledge on infection dynamics during an eight-year period (2015–2023) as well as the clinical, cytological, molecular, and postmortem findings associated with *Haemogregarina stepanowi* infection in a large sample of poached European pond turtles held in Belgrade Zoo quarantine. Cytological and microscopic examination of the samples proved to be sufficient for establishing the infection, but molecular analyses were important in examining the phylogeny of hemoparasites, as well as the association of pathogenicity and the changes they cause in the host. Postmortem findings revealed necrosis and hemorrhages on the shell and skin, followed by massive hemorrhages and inflammatory reactions in the lungs, kidneys, spleen, and liver, along with the presence of parasitic stages of hemogregarines in these organs. Our results of postmortem investigation could be helpful in the future for obtaining the preliminary diagnosis. Finally, it can be hypothesized that the decreased number of diseased and dead turtles over the years can be attributed to the elimination of the leeches as the definitive host through routine pond cleaning and mechanical removal of the leeches. However, the hypothetical role of the birds that land in the vicinity of the pond could not be excluded.

## Data Availability

Data is contained within the article.

## Supplementary Materials

The following supporting information can be downloaded at: https://www.mdpi.com/article/10.3390/ani13152429/s1, Table S1: Average values of hematocrit, hemoglobin, and erythrocyte count for each investigated group.
