# Supplementary material for: Eight-Year Study of Haemogregarina stepanowi Infection in Poached European Pond Turtles (Emys orbicularis) Held in Belgrade Zoo Quarantine
Source: Animals (Basel). 2023 Jul 27;13(15):2429. doi: 10.3390/ani13152429 (PMC10416920; doi:10.3390/ani13152429)
Supplement: Supplementary file 1 [file animals-13-02429-s001.zip › animals-2382084-supplementary.pdf]

Table S1. Average values of hematocrit, hemoglobin and erythrocyte count for each investigated group.

Group	Hematocrit (%)	Hemoglobin (g/dl)	Erythrocyte (x 10^9 cell/ml)
	Referent values 18 - 25	Referent values 5 – 8.4	Referent values 0.5 – 1.5
I (year 2015/2016)*	15.3	4.01	0.4
II (year 2018/2019)**	17	5.5	0.6
III (year 2022/2023)***	14.3	4.5	0.4

*30 animals; ** 9 animals; *** 6 animals
